# Emissions of NO and NH_3_ from a Typical Vegetable-Land Soil after the Application of Chemical N Fertilizers in the Pearl River Delta

**DOI:** 10.1371/journal.pone.0059360

**Published:** 2013-03-19

**Authors:** Dejun Li

**Affiliations:** Department of Microbiology and Plant Biology, University of Oklahoma, Norman, Oklahoma, United States of America; Utrecht University, The Netherlands

## Abstract

Cropland soil is an important source of atmospheric nitric oxide (NO) and ammonia (NH_3_). Chinese croplands are characterized by intensive management, but limited information is available with regard to NO emissions from croplands in China and NH_3_ emissions in south China. In this study, a mesocosm experiment was conducted to measure NO and NH_3_ emissions from a typical vegetable-land soil in the Pearl River Delta following the applications of 150 kg N ha^−1^ as urea, ammonium nitrate (AN) and ammonium bicarbonate (ABC), respectively. Over the sampling period after fertilization (72 days for NO and 39 days for NH_3_), mean NO fluxes (± standard error of three replicates) in the control and urea, AN and ABC fertilized mesocosms were 10.9±0.9, 73.1±2.9, 63.9±1.8 and 66.0±4.0 ng N m^−2^ s^−1^, respectively; mean NH_3_ fluxes were 8.9±0.2, 493.6±4.4, 144.8±0.1 and 684.7±8.4 ng N m^−2^ s^−1^, respectively. The fertilizer-induced NO emission factors for urea, AN and ABC were 2.6±0.1%, 2.2±0.1% and 2.3±0.2%, respectively. The fertilizer-induced NH_3_ emission factors for the three fertilizers were 10.9±0.2%, 3.1±0.1% and 15.2±0.4%, respectively. From the perspective of air quality protection, it would be better to increase the proportion of AN application due to its lower emission factors for both NO and NH_3_.

## Introduction

Increases of atmospheric trace gases are of increasing concern due to their roles in detrimental health and environmental effects. Nitrogen oxides (NO_x_ = NO+NO_2_) and ammonia (NH_3_) are among these gases. NO_x_ catalyze the photochemical formation of ground-level ozone [1], which is a potent greenhouse gas [2] and poses a threat to human health and vegetation on regional scales [3]. The photochemical end product, HNO_3_, is a major component of acid rain [1]. NH_3_ enhances aerosol formation [4], and hence influences regional air quality.

Soil is a major source for atmospheric NO_x_. According to Davidson and Kingerlee (1997), the source strength of soils is just inferior to fossil fuel combustion globally, while cropland is the second largest contributor to atmospheric NO_x_ among all types of land uses. Current estimates of soil NO emissions from cropland range from 2.4–5.4 Tg N yr^−1^ [5], [6], [7]. The large uncertainty is largely caused by limited data availability, especially in tropical and subtropical regions. Furthermore, fertilized cropland (12.6 Tg N yr^−1^) ranks second among all the sources of atmospheric NH_3_ and is responsible for 23.5% of the global annual emission [8]. Of the emitted NO and NH_3_ from cropland, the major part is induced by fertilizer application [9].

Chinese croplands have been intensively managed. With the cropland area only occupying 7% of the global total, the annual consumption of synthetic N fertilizer in China accounted for ca. 30% of the total global consumption in 2004 [10]. The annual fertilizer N consumption in China has been increasing by about 6.5%, which is higher than the mean rate of 4.8% for other Asian countries [11]. Urea (CO(NH_2_)_2_) and ammonium bicarbonate (NH_4_HCO_3_, ABC) are the two most widely used synthetic N fertilizers in China, with the latter only used in China [9], [12].

**Figure 1:**
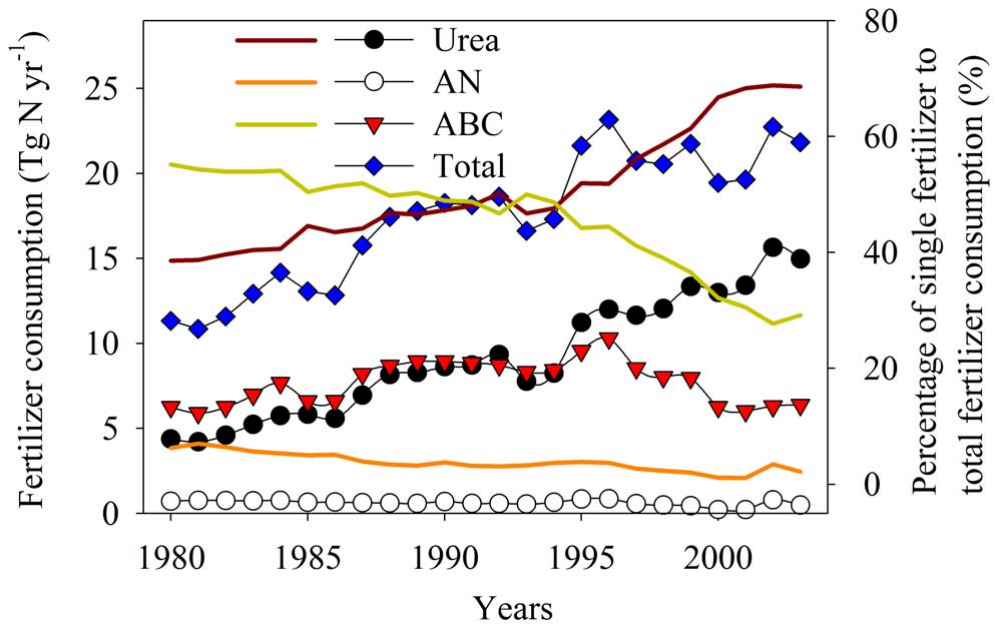
Changes of fertilizer consumption (line and scatter) and percentage (bold line) of single fertilizer consumption to total consumption of urea, ammonium nitrate (AN) and ammonium bicarbonate (ABC) during 1980 to 2003 in China [10].

Most of the previous studies on NH_3_ volatilization from Chinese croplands were carried out in North China Plain and eastern China’s Yangtze River Delta [12], but, to our knowledge, little information is available for south China. NO emissions from Chinese croplands have been reported by several studies [11], [13], [14], [15], [16], [17], [18]. However, no study has been conducted to investigate NO emission following ABC application, although ABC is a main N fertilizer in China (Figure 1), Due the lack of a NO emission factor for ABC, one study used the emission factor for ammonium sulphate as an estimate of that for ABC [19].

Vegetable land forms an important part of China’s croplands. On a national scale, vegetable land accounts for ca. 7% of the total cropland, and the ratios are typically higher in developed regions [15]. For example, in south China’s Guangdong province, the ratio is 24.2% (1.16×10^6^ ha) in 2005 [15]; in the Yangtze Delta of east China, vegetable land (ca. 6×10^6^ ha) also occupies one fifth of the total cropland area [14]. Compared with other cultivated lands, vegetable lands receive more fertilizer-N per unit of area. For example, N application rates in individual growing season for vegetables range from 300 to 700 kg N ha^−1^, compared to 150–300 kg N ha^−1^ for non-vegetable crops [20]. These higher N fertilizer application rates to vegetable lands would inevitably induce larger amount of N loss to the environment, including leaching of nitrate to surface/ground water, and emission of nitrogen-containing gases to the atmosphere.

In the present study, we conducted a mesocosm experiment in which we measured emissions of NO and NH_3_ from a subtropical vegetable-land soil following the application of urea, ABC, and NH_4_NO_3_ (AN), respectively. The main objective was to quantify the emission factors for the three synthetic fertilizers.

## Materials and Methods

### Ethics Statement

The vegetable field for arable soil sampling was privately owned. We collected soil under the permission of the land owner Mrs Cheng. All other necessary permits were obtained for the described field studies.

### Site Description

The experiment was conducted at a suburban site (23°10'N, 113°23'E) of Guangzhou, the capital of Guangdong province. This area enjoys a subtropical monsoon climate, with an annual mean rainfall of 1938.2 mm and annual mean air temperature of 22.5°C. Monthly mean air temperature is lowest in January (13.3°C) and highest in July (29.5°C) [21].

Flowering Chinese cabbage (*Brassica campestris* L. ssp. *Chinensis* var. *utilis* Tsen et Lee) is the most widely cultivated vegetable type in Guangdong Province [15]. Vegetables can grow under field conditions all year round in this region. Typically, a new batch of vegetables is planted approximately five days after a batch of vegetables is harvested, and thus no long fallow period exists. At the study site, a typical cultivation cycle for flowering Chinese Cabbage takes about 47 days and mainly involves five management events: sowing, harrowing and transplanting, first fertilization, second fertilization, harvesting and harrowing. The rate of fertilizer application is about 320 kg N ha^−1^ yr^−1^ with about 5 batches of vegetables grown in a year according to our previous field study [15].3

### Experimental Design

The arable soil (top layer of 20 cm) used in this study was collected from a vegetable field which has been cultivated for flowering Chinese cabbage for more than 10 years (Personal communication with the farmer). The soil type is lateritic red earth, which is typical in subtropical China. Soil properties (mean ± SE, n = 5) were analyzed after the soil was well mixed. Soil pH (extracted with KCl solution), contents of total C and total N were 6.99±0.02, 1.69±0.07% and 0.14±0.01%. Soil texture was sandy clay loam with a bulk density of 1.18±0.18 g cm^−3^ at the mid of a cultivation cycle of the vegetable [22].

On December 10, 2005, mixed soil (after excluding coarse roots and residuals) was put into twelve plastic containers, each with a height of 40 cm and an area of 1 m^2^. There was a 10-cm layer of gravel and 10 holes (each with a diameter of about 0.5 cm), which were used for drainage, at the bottom of each container. Each container was filled with 362 kg soil (with gravimetric soil water content of about 18.5%) to a depth of 25 cm to achieve the field bulk density measured at the mid of a cultivation cycle. Therefore, our study can be regarded as a mesocosm experiment. The mesocosms were placed on an open field in our institute, at a distance of approximately 2 km from the vegetable fields.

Urea, AN and ABC were applied at a rate of 150 kg N ha^−1^. Each treatment had three replications. On December 29, 2005 (the temperature of this period was similar to the annual average, details are shown in the following section), the fertilizers were dissolved in 1 L water and applied to the soil surface with a handhold sprayer. Three mesocosms were used as control and was only treated with 1 L water. The treatment process completed within 30 min. Deionized water was sprayed to the soil to maintain the soil moisture within a range similar to that of the field (with gravimetric water content of 16.6%–22.5%, which are equivalent to volumetric soil water content of 19.6–26.6% based on the relationship between gravimetric water content and volumetric water content [23]) [15] by using moisture probe meter (MP-508B, China) to monitor the change of volumetric soil water content at the depth of 10 cm.

### Measurement of NO Fluxes

Measurement of NO fluxes started on December 25, 2005 and lasted until March 10, 2006. Measurements were conducted every one day before fertilization. Upon fertilization, NO fluxes were measured once a day for a week and then once every two days for the following two weeks. Thereafter, measurement was conducted once every 4–12 days. On each sampling day, flux measurements were conducted in the morning (9∶30–10∶30), at noon (12∶00–13∶00), and in the afternoon (3∶00–4∶00).

A dynamic flow-through chamber technique was used to measure NO fluxes. The chamber system was described in detail previously [24], [25]. The chambers are made of stainless steel (inner walls coated with Teflon films), each covering an area of 30 cm ×30 cm with a total volume of 9 L. Each chamber has one inlet port, one exhaust port and one outlet port for sampling. Inside each chamber, a thermo-sensor is fixed to measure air temperature, and a fan is attached to ensure sufficient mixing of air within the chamber. Different from the chamber system described previously [24], [25], for this study each chamber was coupled with a steel pedestal (with a depth of 10 cm), at the top of which was a water-filled channel. One pedestal was put in the center of each container and inserted into the soil with a depth of 10 cm. During sampling, water was added to the channel of the pedestal so that the chamber and pedestal were sealed by the water. An additional reference chamber, closed at the bottom with Teflon sheet, was employed for in situ quantification of chemical reactions and chamber wall deposition effects. Ambient air was pumped into the chambers at a rate of 4 L min^−1^ through 10 m long Teflon tubes with inner diameters of 4.8 mm, and the sample air was taken in through tubes of the same dimension. The residence time of air in the chambers was about 2.25 min. After about 15 min (over 5 cycles of residence time) when a steady state was reached inside the chambers, NO was analyzed by a model 42C chemiluminescence NO-NO_2_−NO_x_ analyzer (Thermo Environmental Instruments Inc., USA) for 3 minutes. By the difference of sampling chambers and the reference chamber, net fluxes from the soils could be calculated from equation (1) [26]:
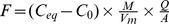
(1)where F is the net flux in ng m^−2^ s^−1^, M is the atomic weight of the element (N = 14.008 g mol^−1^, C = 12.011 g mol^−1^), V_m_ is the standard gaseous molar volume (24.055×10^−3^ m^−3^ mol^−1^), C_eq_ is the mixing ratio (ppbv = 10^−9^ m^−3^ m^−3^) of the gas when the chamber under consideration has reached steady state, C_0_ is the mixing ratio of the gas in the reference chamber, Q is the mass flow rate of air through the chamber (0.667×10^−5^ m^3^ s^−1^), and A is the soil surface area (0.09 m^2^) covered by the chamber.

### Measurement of NH_3_ Fluxes

Measurement of NH_3_ fluxes started on December 25, 2005 and lasted until February 4 2006. After sampling of NO fluxes, NH_3_ was sampled with an acid trap method, which is commonly used for NH_3_ measurement [27]. During sampling, the flow rate was also 4 L min^−1^, and sample air was drawn at a rate of 1 L min^−1^ for 30 min to the bubblers containing 20 ml of 0.7 M H_2_SO_4_. The NH_4_^+^ concentration trapped in the acid was determined by the indophenol blue method. Results gained with the acid trap method have been proven to be comparable with those by chemiluminescence NH_3_−NO_x_ analyzer [27]. Net fluxes were calculated by the difference of sampling chambers and the reference chamber using the equation (2):
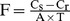
(2)where C_s_ and C_r_ represent the amount of N (ng) trapped in the bubblers connected to the sampling chamber and reference chamber, respectively. A is the soil surface area (0.09 m^2^) covered by the chamber, and T is the sampling time (1800 s).

### Auxiliary Measurements

Along with measurement of fluxes, air temperature at a height of 1.5 m, temperature inside the chambers, and soil temperature at 0–5 cm depth was measured with soil temperature probes (TES Ltd., China).

At each sampling date, three composite soil samples were collected from 0–5 cm depth. These samples were used for determinations of gravimetric soil moisture and KCl-extractable inorganic nitrogen pools. Briefly, 40 g fresh soil samples were extracted with 200 ml KCl solution (1 mol L^−1^) for one hour. NH_4_^+^-N was measured by indophenol blue method and determined spectrophotometrically at 630 nm; NO_3_^–^N was determined by reduction to nitrite (NO_2_−N) via a cadmium reactor, diazotized with sulfanilamide and is coupled to N-(1-Napthyl)-ethylenediamine dihydrochloride to form an azochromophore measured spectrophotometrically at 543 nm [22].

### Statistics and Data Analysis

Daily averaged flux of NO or NH_3_ for each mesocosm was the average of the three measurements on each sampling day. Linear interpolation approach was used to estimate the daily fluxes for dates on which measurements were not made based on the fluxes from the dates when measurements were conducted immediately preceding and immediately following the dates that were not measured. Mean fluxes (ng N m^−2^ s^−1^) over the sampling period after fertilization (72 days for NO and 39 days for NH_3_) were calculated after data interpolation. Total emissions (E_total_, mg N m^−2^) were calculated by multiplying mean fluxes by sampling days after fertilization. Net emission (E_net_, mg N m^−2^) was obtained by subtracting E_total_ of the control from that of the corresponding fertilized mesocosms. The emission factor (%) of NO (F_NO_) or NH_3_ (F_NH3_) was calculated based on N atoms according to equation (3):
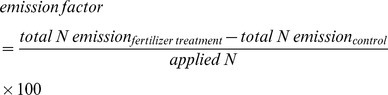
(3)


Reported data of each treatment were the mean of the three replicate mesocosms on a daily basis. ANOVA analyses with post hoc LSD tests were performed using SPSS 10.0 (SPSS Ltd., USA) to identify differences among treatments. In this paper, analyses with *P* values <0.05 were considered significant.

## Results and Discussion

### NO Fluxes

**Figure 2:**
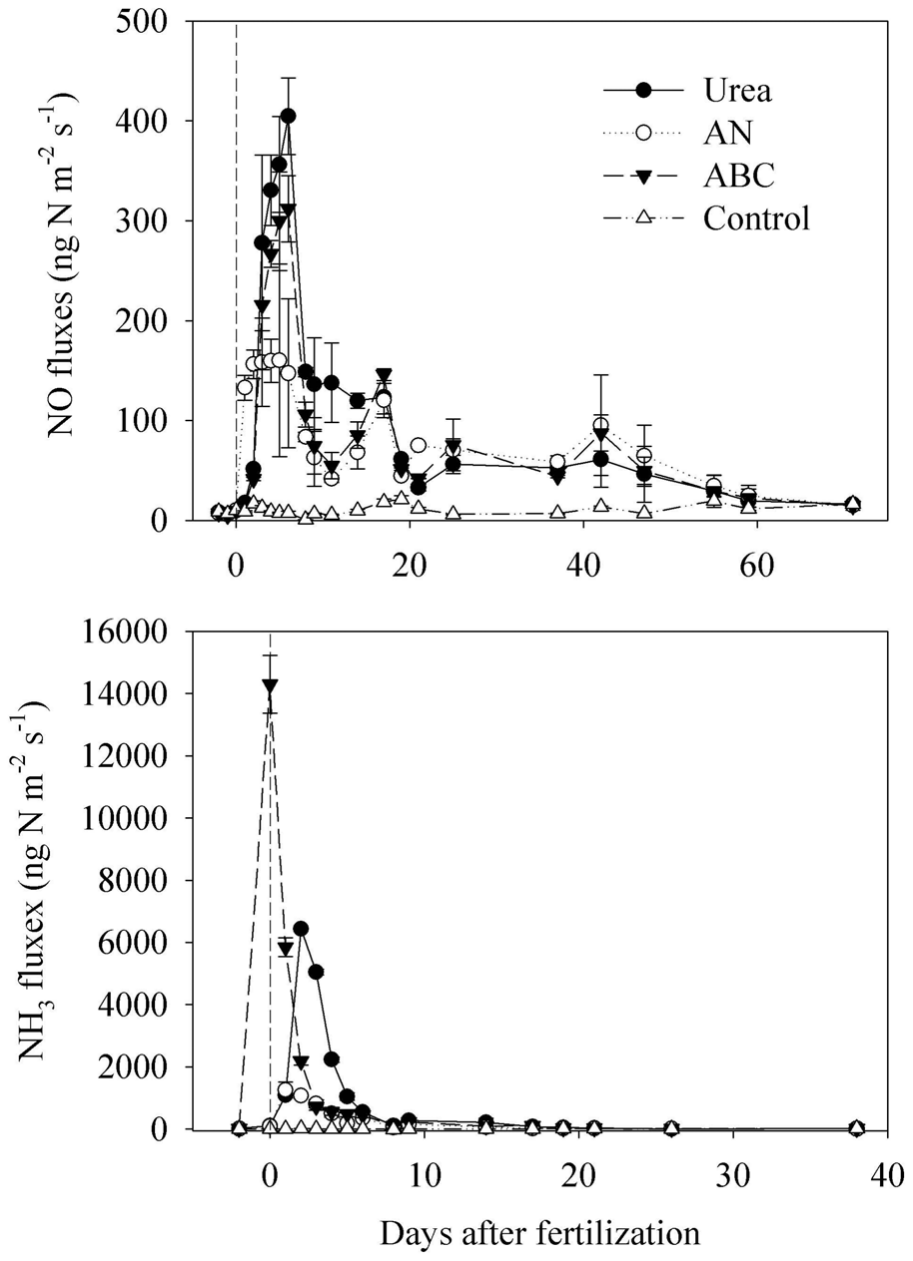
NO and NH_3_ fluxes during the sampling period. Each value is the mean of three replicates, and error bars represent standard errors. Vertical dash line indicates the day when fertilizers were applied.

Before fertilization, the mean NO flux was 6.9±0.6 (mean ± standard error of three replicates) ng N m^−2^ s^−1^ (Figure 2). The NO flux increased quickly in the AN fertilized mesocosms and reached 132.8±12.4 ng N m^−2^ s^−1^ one day after fertilization. The peaks of NO fluxes for different fertilizers occurred at different times. For AN fertilized mesocosms, the flux peaked 5 days after fertilization. For both the urea and ABC fertilized mesocosms, flux peaks were observed 6 days after fertilization. However, the peak fluxes for different fertilizers varied greatly. The averaged peak flux for urea was the greatest (404.9±38.3 ng N m^−2^ s^−1^), much higher (*P*<0.05) than that for AN (160.4±96.5 ng N m^−2^ s^−1^) or for ABC (312.1±33.0 ng N m^−2^ s^−1^) (Figure 2). The enhancement effects of fertilization on NO emissions lasted nearly two months.

Over the experimental period after fertilization, mean NO fluxes were 10.9±0.9, 73.1±2.9, 63.9±1.8 and 66.0±4.0 ng N m^−2^ s^−1^, respectively, for the control, urea, AN and ABC treatments (Table 1). The averaged NO flux for urea was significantly greater than those for AN and ABC (*P*<0.05). We previously reported that NO fluxes in a typical vegetable field (planted with flowering Chinese cabbage), where the soil for the current study was collected, varied from 20.0 to 122.1 ng N m^−2^ s^−1^ with an average of 47.5 ng N m^−2^ s^−1^ [15], which was relatively small compared with the fluxes in the present study. However, considering that only 63.5 kg N ha^−1^ was applied in the field study (also see Table 2), a lower averaged NO flux was reasonable. According to a review paper, NO emissions ranged from 1.19 to 44 ng N m^−2^ s^−1^ from recently fertilized agricultural soils in temperate regions [5]. However, surprisingly high NO fluxes were also recorded in temperate croplands [28]. For example, Boeckx and Van Cleemput [28] observed that NO fluxes in autumn of 1997 (fertilized with 144 kg N ha^−1^) and spring of 1998 (fertilized with 88 kg N ha^−1^ as cattle slurry) and summer of 1998 (fertilized with 122 kg N ha^−1^ as chicken manure) were19, 68, and 581 ng N m^−2^ s^−1^, respectively, from sandy arable soil in Belgium. Measurements of NO fluxes in tropical croplands were rather limited, but the available fluxes from tropical croplands were not significantly higher than those from temperate croplands [5], suggesting that the effect of latitude was small.

**Table 1:** Fluxes of NO and NH_3_, total emissions (E_total_), net emissions (E_net_), and emission factors (EF) for the three fertilizers.

	Flux	E_total_	E_net_	EF
	ng N m^−2^ s^−1^	mg N m^−2^	mg N m^−2^	%
NO				
Urea	73.1±2.9 a	454.6±17.9 a	386.7±18.7 a	2.6±0.1 a
AN	63.9±1.8 b	397.5±10.9 b	329.6±12.2 b	2.2±0.1 b
ABC	66.0±4.0 b	410.3±25.1 b	342.4±25.7 b	2.3±0.2 b
Control	10.9±0.9 c	67.9±5.5 c		
NH_3_				
Urea	493.6±4.4 b	1663.1±27.3 b	1633.2±27.3 b	10.9±0.2 b
AN	144.8±0.1 c	488.0±0.5 c	458.1±1.1 c	3.1±0.1 c
ABC	684.7±8.4 a	2307.1±52.2 a	2277.2±52.2 a	15.2±0.4 a
Control	8.9±0.2 d	29.9±1.0 d		

Values are presented as mean ± standard error of three replicates. Different letters in a column denote that the mean values are significantly different at *P*<0.05 level.

**Table 2:** NO emissions from fertilized upland arable fields in China.

Location	Land-use type	Fertilizer type	Duration	N rates	NO flux	Emission factors %
				kg N ha^−1^	ng N m^−2^ s^−1^	
39°57'N; 116°18'E^a^	Corn	OM^j^	N/A	88.5	0.14	0.04
39°57'N; 116°18'E^a^	Corn	Urea	N/A	150	77.5	1.6
39°57'N; 116°18'E^a^	Corn	Urea	N/A	300	76.4	0.75
39°57'N; 116°18'E^a^	Corn	OM+urea	N/A	238.5	66.4	0.78
39°57'N; 116°18'E^a^	Corn	OM+urea	N/A	238.5	150.8	1.3
34°56'N; 110°43'E^b^	Cotton	Urea+DP+PS	Jan-Dec	66.3	2.4	0.24
32°35'N; 119°42'E^c^	Bare soil	Urea	Jan-Dec	153	70.4	0.67
32°35'N; 119°42'E^c^	Vegetables*		Jan-Dec	118–548	2.5–142.7	0.05–1.24
31°16'N; 120°38'E^d^	Wheat	M+CF+urea	Nov-Jun	191	21.3	1.75
31°16'N; 120°38'E^d^	Wheat	CF+urea	Nov-Jun	191	28.5	2.5
31°16'N; 120°38'E^d^	Wheat	CF+urea	Nov-Jun	191	22.4	1.87
30°50'N; 120°42'E^e^	Cabbage	CF	Mar -Jun	45	11.5	0.6
30°50'N; 120°42'E^e^	Potato	CF+M	Mar -Jun	45.6	34.2	3.6
30°50'N; 120°42'E^f^	Cabbage	CF	Mar -Jun	135	20.9	1.05
30°50'N; 120°42'E^f^	Potato	CF+ M	Mar -Jun	108	27.4	1.75
30°50'N; 120°42'E^f^	Soybean	CF+urea	Mar -Jun	81	21.4	1.83
30°50'N; 120°42'E^g^	Cabbage	CF+M+urea	Aug-Dec	271.2	33.8	1.2
30°50'N; 120°42'E^g^	Garlic	CF+M+urea	Aug-Dec	267.3	360	11.56
30°50'N; 120°42'E^g^	Radish	CF+M+urea	Aug-Dec	263.6	76	2.56
23°10'N; 113°23'E^h^	FCC	CF+M+urea	Sep-Oct	63.5	47.5	2.4
23°10'N; 113°23'E^i^	Bare soil	Urea	Dec-Mar	150	73.1	2.6
23°10'N; 113°23'E^i^	Bare soil	AN	Dec-Mar	150	63.9	2.2
23°10'N; 113°23'E^i^	Bare soil	ABC	Dec-Mar	150	66	2.3

a Walsh [37];

b Liu et al. [18];

c Mei et al., [16];

d Zheng et al. [11];

e Fang and Mu [14];

f Fang and Mu [38];

g Pang et al. [17];

h Li and Wang [15];

i This study.

j OM–organic matter; CF–compound fertilizer; M–Manure; FCC– Flowering Chinese cabbage; DP-diammonium phosphate; PS-potassium sulphate;

* Different vegetables were cultivated from 2004 to 2008 and various fertilizers were used.

N/A: not available.

Chinese croplands are famous for relatively high N fertilization. Table 2 compiles the available data of NO fluxes from Chinese cropland soils. Averaged NO fluxes over the sampling periods varied from 0.14 to 360 ng N m^−2^ s^−1^. There is a strong correlation between N fertilizer rates and NO fluxes in an exponential way (y  = 6.7861e^0.0094x^, R^2^ = 0.27, *P*<0.05, n = 23). However, there is no significant correlation between latitude and NO fluxes across these studies (*P*>0.05, Table 2). Our results confirm that there is no strong effect of latitude on NO fluxes.

### Influencing Factors for NO Fluxes

**Figure 3:**
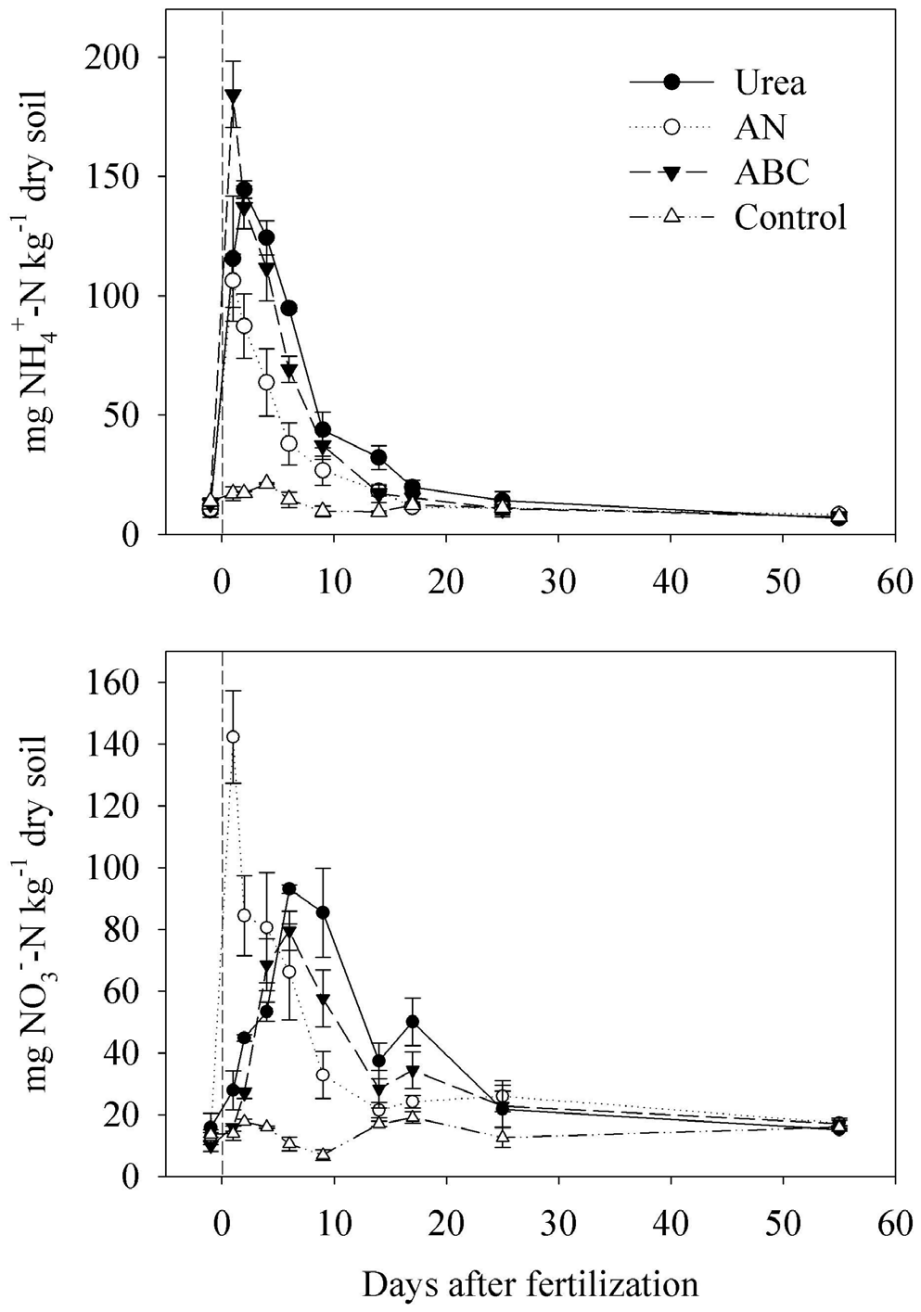
Soil inorganic N pools during the sampling period. Each value is the mean of three replicates, and error bars represent standard errors.

**Figure 4:**
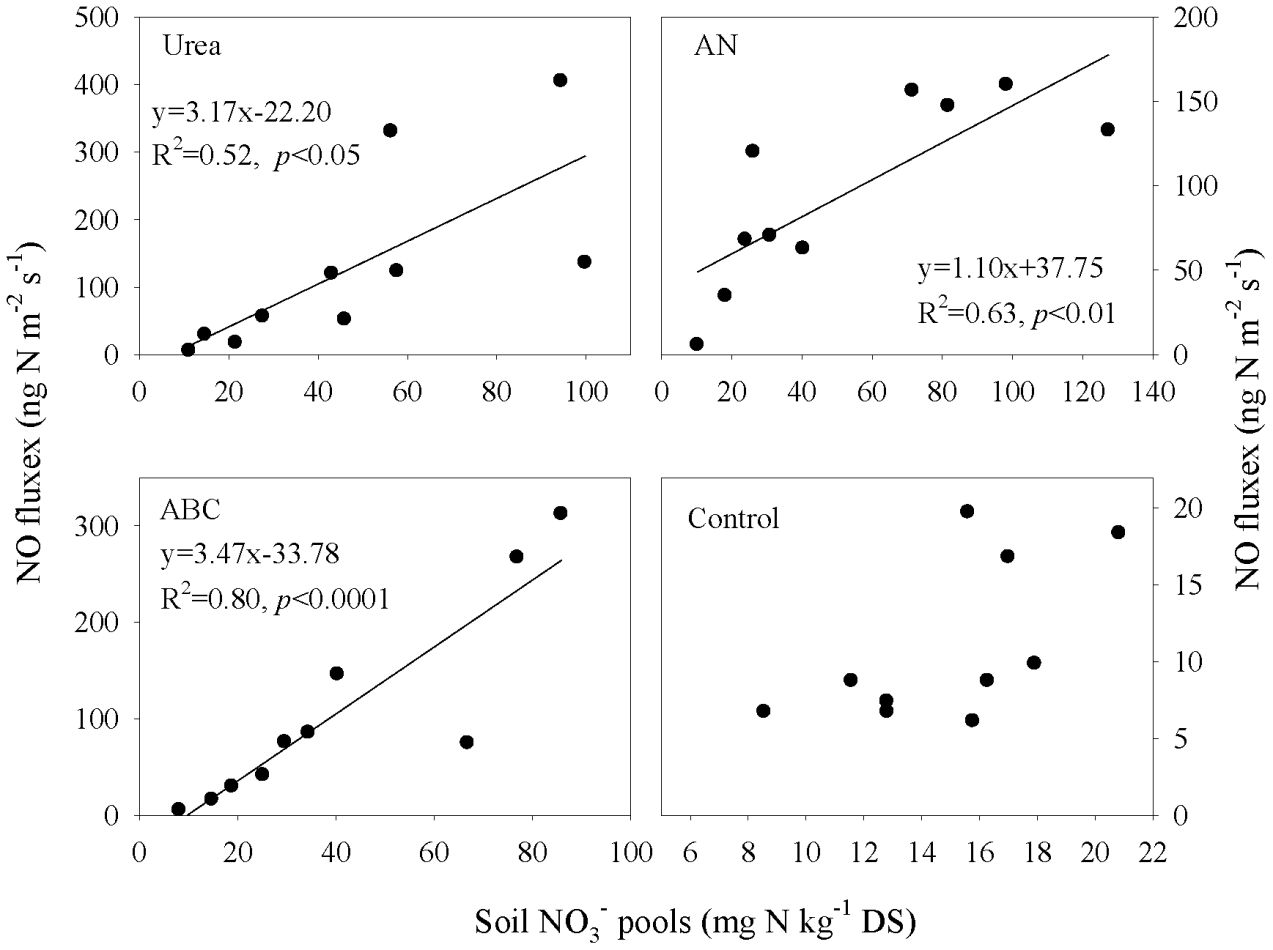
Correlation between NO fluxes and soil NO_3_^−^ pools (n = 10). Each value is the mean of three replicates.

Many factors influence soil NO emissions, including N availability, soil moisture, soil temperature, atmospheric NO concentration, and other environmental factors that regulate the underlying processes of NO production and consumption in soils [29]. In well-drained agricultural soils, application of N fertilizers is often observed to cause large NO emissions [30]. This is because N fertilization supplies substrates (NH_4_^+^ and NO_3_^−^) to nitrifying or denitrifying bacteria, which are responsible for soil biogenic NO production [29]. In the current study, soil NO_3_^−^ pools increase after NH_4_^+^-based fertilizer application, indicating the occurrence of nitrification (Figure 3). Soil NH_4_^+^ pools were found to be significantly correlated with NO emissions only in AN fertilized mesocosms (*R* ^2^ = 0.60, *p*<0.01), but soil NO_3_^−^ pools were significantly correlated with NO emissions in all fertilized mesocosms (Figure 4). Since NO_3_^−^ is the product of nitrification and substrate of denitrification, it is difficult to judge which process is mainly responsible for NO production from the correlation of NO_3_^−^ and NO fluxes in the current study. Nevertheless, our field study indicated that nitrification was the dominant process [15]. Since the soil used in the current study was collected from the same vegetable field where our field study was conducted, nitrification was probably also the main process responsible for NO production in the current study [15].

**Figure 5:**
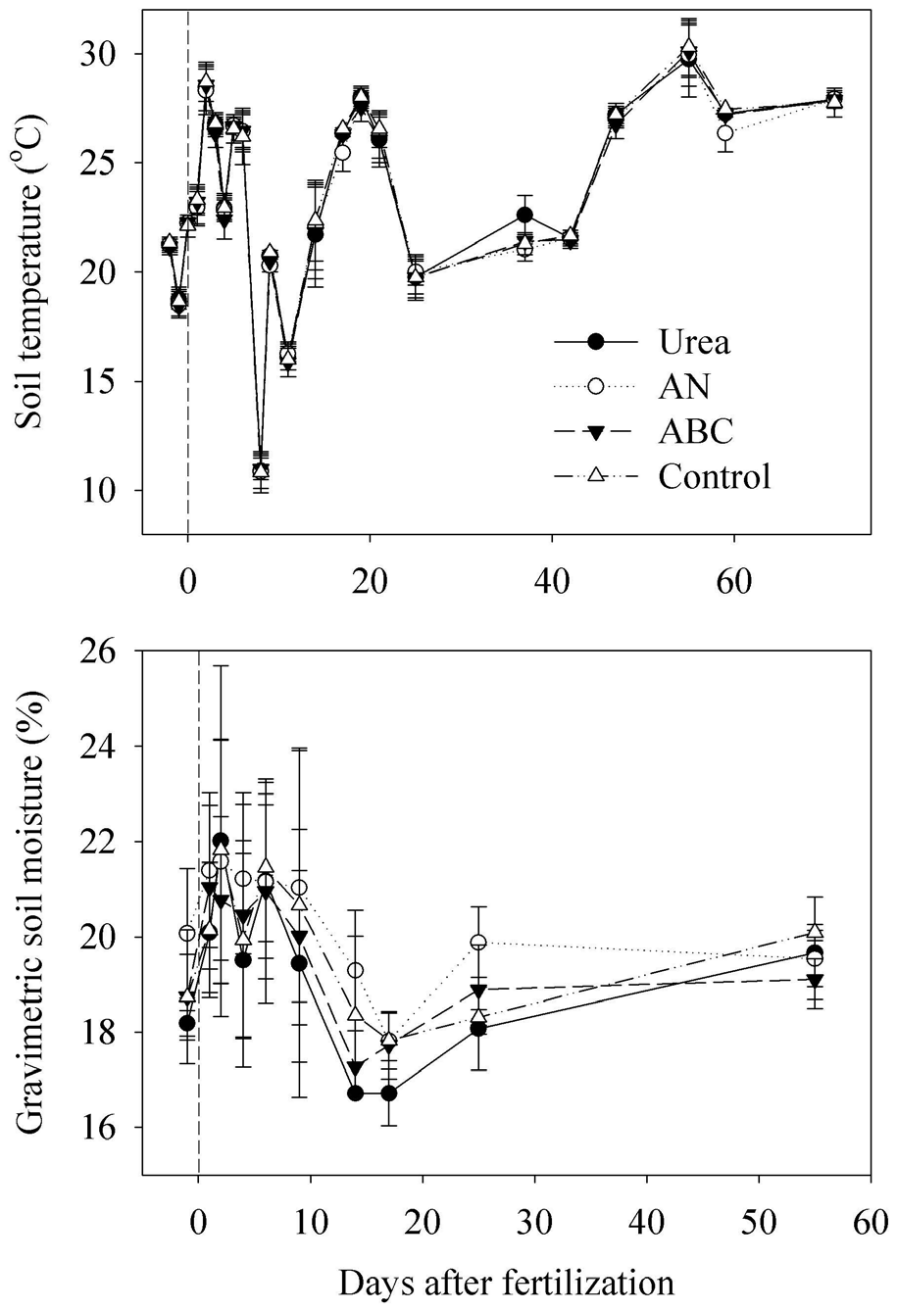
Variation of soil temperature and soil moisture in different treatment mesocosms during the sampling period. Each value is the mean of three replicates, and error bars represent standard errors. Vertical dash line indicates the day when fertilizers were applied.

Soil temperature and moisture as important regulators on NO emissions have often been reported [11], [15], [24], [29]. In the present study, soil temperature at 0–5 cm depth ranged between 10–30°C (Figure 5). A strong correlation was only found in the control (F = 0.46exp^0.12T^, *R* ^2^ = 0.62, *P*<0.0001, n = 23), probably because the effects of fertilization masked the effects of soil temperature in the fertilized mesocosms. Soil moisture (Figure 5) was maintained within a narrow range (16–25%) during the experiment, which was similar to the field soil moisture (16.6–22.5%) of typical vegetable land in this region [15]. No clear correlation was found between NO fluxes and soil moisture, probably because during the experimental period of this study, soil moisture varied within a narrow range.

### NH_3_ Fluxes

Before fertilization, the average NH_3_ flux was 10.7±3.8 ng N m^−2^ s^−1^. NH_3_ volatilization from the ABC fertilized mesocosms increased sharply immediately after fertilization, and reached a maximum on the treatment day, then decreased rapidly (Figure 2). NH_3_ flux from urea fertilized mesocosms peaked 3 days after fertilization (Figure 2). Peak values of NH_3_ fluxes from AN fertilized mesocosms were observed 2 days after fertilization (Figure 2). The enhancement effect of fertilization on NH_3_ fluxes lasted nearly three weeks. Our results were consistent with others in the pattern of changes of NH_3_ volatilization following fertilization, i.e., ABC usually led to large NH_3_ volatilization immediately after application, then reduced dramatically [31]. Urea is not volatile itself, but will become volatile after it is hydrolyzed into NH_4_^+^ and CO_2_ by ureases in the soil. This process was found to be completed within 2–3 days, and NH_3_ volatilization increases with increasing pH and temperature [8].

NH_3_ flux measurements lasted 39 days after fertilization. During this period, mean NH_3_ fluxes from urea, AN and ABC fertilized mesocosms were 493.6±4.4, 144.8±0.1 and 684.7±8.4 ng N m^−2^ s^−1^, respectively, compared to 8.9±0.2 ng N m^−2^ s^−1^ from the control (Table 1). The difference of averaged fluxes between any two fertilizers were significantly different (*P*<0.5).

### N loss as NO

Total NO emissions from urea, AN and ABC fertilized mesocosms were 454.6±17.9, 397.5±10.9, 410.3±25.1 mg N m^−2^, respectively, significantly greater than that from the control (*P*<0.05) (Table 1).

With regard to fertilizer-induced NO emission factor (EF), there was no significant difference between AN and ABC (*P*>0.05), but EF for urea (2.6±0.1%) was significantly greater (*P*<0.05) than the other two fertilizers (Table 1). Our data supported that urea had the highest EF [32], but was in conflict with some studies which reported that a mixed form fertilizer such as AN had the greatest emission in tropical savanna soil [33]. FAO/IFA [19] used EFs for urea, AN and ABC of 0.6%, 0.5% and 0.8%, respectively. However, they used the EF for ammonium sulphate as an estimate of EF for ABC since no study was conducted to determine EF for ABC at that time.

The fertilizer-induced NO emission factors based on studies in fertilized upland arable fields in China were presented in Table 2. The field measured EFs varied from 0.04 to 11.56 with an average of 1.93 (excluding EFs from the current study). The EFs observed in the present study were within the reported range but greater than the average of all the filed observed values. However, the EFs in the current study are quite similar to those derived from the field study (EF = 2.4%) conducted at a nearby site [15]. Nevertheless, it should be noted that the EFs in the current study ware based on a mesocosm experiment, therefore limitations may be involved when making comparison with field based studies.

### N Loss as NH_3_

Total emission of NH_3_ from ABC fertilized mesocosms was the highest (2.31 g N m^−2^), which was 1.4 and 4.7 times that from urea and AN fertilized mesocosms, respectively, and 77 times that from the control (*P*<0.05, Table 1). Total emission of NH_3_ was significantly greater from urea than from AN fertilized mesocosms (*P*<0.05).

Fertilizer-induced NH_3_ emission factors varied greatly for the three fertilizers (Table 1). EF for ABC was 15.2±0.4%, which was 1.4 times that for urea (10.9±0.2%), and 5 times that for AN (3.1±0.1%). Similarly, some studies in China reported that EF for ABC was about 1.5 times that for urea [34], [35]. Yan et al. (2003) estimated NH_3_ emission from dry croplands in East, South East and South Asia by adopting emission factors of 13.7% for urea and 20.5% for ABC. As no emission data for AN were available from Asian croplands, Yan et al. (2003) adopted an EF of 2.0%, which was proposed by European Environment Agency. According to the present study, the adoption of the European proposed EF for AN likely only slightly underestimated NH_3_ emission due to AN fertilization in China. Nevertheless, more studies are needed since only one cropland soil was investigated in the current study and since our study was conducted as a mesocosm experiment.

It must be stressed that the chamber, though widely used, may lead to NH_3_ volatilization rates somewhat different from those under field conditions. Wind speed exerts positive effects on NH_3_ volatilization loss [36], but air currents inside the chamber by flowing through of air in addition to the mixing by fans, can not well simulate the wind above the soil under natural conditions. Thus as an approximation, NH_3_ volatilization measured in this study might not be always consistent with that under field conditions.

### Implications for National Emissions of NO and NH_3_

In China, urea and ABC are two main N fertilizers, while AN only accounts for a small portion in total fertilizer N consumption. During 1980 to 2003 (Figure 1), urea consumption increased continuously; ABC consumption increased until 1996 and decreased thereafter; AN consumption tended to decrease continuously [10]. The total consumption of fertilizer N (only for the three fertilizers) peaked in 1996 and then levelled off (Figure 1). The portion of urea consumption increased from 38.6% to 68.6% of the total consumption of the three fertilizers, but that of ABC and AN decreased accordingly from 55.2% to 29.2% and from 6.3% to 2.2%, respectively (Figure 1). Since the emission factors of both NO and NH_3_ are different for the three fertilizers, changes in fertilizer consumption and proportion would have a substantial impact on national budgets of NO and NH_3_. Since the current study compars the emission factors for the three fertilizers from a typical cropland soil in China, the impact of changes in fertilizer consumption and proportion on national budgets of NO and NH_3_ can be roughly estimated. However, since only one soil type was studied using a mesocosm experiment design, there are limitations involved in these estimates.

**Figure 6:**
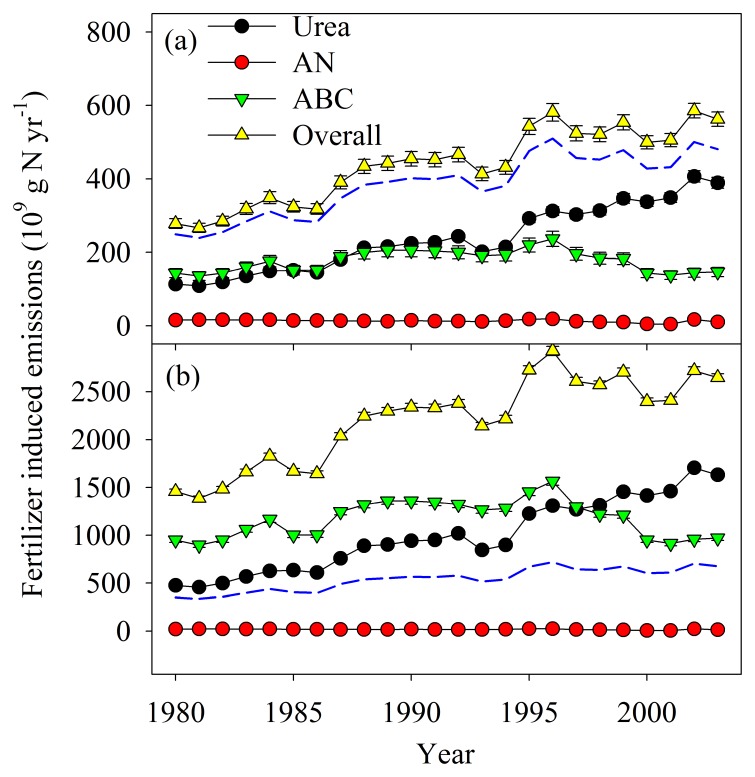
Changes of fertilizer induced emissions of (a) NO and (b) NH_3_ by consumption of urea, ammonium nitrate (AN) and ammonium bicarbonate (ABC) in China between 1980 and 2003. Fertilizer induced emissions of NO and NH_3_ were calculated by multiplying annual consumption of each fertilizer (Figure 1) by the corresponding emission factor obtained in the current study. The blue line in each panel represents the total emission of NO or NH_3_ if the other two fertilizers were replaced by AN.

Total emissions of both NO and NH_3_ from the three fertilizers peaked in 1996 and then levelled off (Figure 6). But for different fertilizers, the changes of emission during 1980–2003 are different. Emissions from urea application increased continuously for both gases. Emissions from ABC application peaked in 1996 and then decreased. Emissions from AN application were much smaller compared to the other two fertilizers and decreased over the period. Urea application has been the dominant source since 1994 for NO and since 1998 for NH_3_. Since EFs of both NO and NH_3_ emissions were the lowest for AN among the three studied fertilizers, the complete replacement of the other two fertilizers by AN would greatly decrease NO and NH_3_ emissions, especially NH_3_ (Figure 6). Therefore, in order to reduce air pollution, it would be better to increase the proportion of AN application.

### Conclusions

In the present study, a mesocosm experiment was conducted to measure NO and NH_3_ emissions from bare soil, which was collected from vegetable land, following the application of urea, AN and ABC. Emission factors of NO were 2.6%, 2.2% and 2.3% for the above three fertilizers, respectively; and emission factors of NH_3_ were 10.9%, 3.1% and 15.2%, respectively. From the perspective of air quality protection, it would be better to increase the proportion of AN application due to its lower emission factors for both NO and NH_3_.
